# Risk factors for pulmonary cement embolism after percutaneous vertebroplasty and radiofrequency ablation for spinal metastases

**DOI:** 10.3389/fonc.2023.1129658

**Published:** 2023-05-05

**Authors:** Luqiang Wang, Ming Lu, Xinxin Zhang, Zhenguo Zhao, Xiaoyang Li, Ting Liu, Libin Xu, Shengji Yu

**Affiliations:** ^1^ Department of Orthopedics, National Cancer Center/National Clinical Research Center for Cancer/Cancer Hospital, Chinese Academy of Medical Sciences and Peking Union Medical College, Beijing, China; ^2^ Department of Endocrinology, Beijing Tongren Hospital, Capital Medical University, Beijing, China

**Keywords:** spinal metastases, pulmonary cement embolism, PVP, RFA - radiofrequency ablation, cement vein leakage

## Abstract

**Objective:**

Pulmonary cement embolism is a rare but underestimated complication of vertebroplasty due to the relative lack of study and examination. This study aims to investigate the incidence of pulmonary cement embolism in patients with spinal metastasis who undergo PVP with RFA and to analyze the relative risk factors.

**Methods:**

A total of 47 patients were retrospectively included and classified into pulmonary cement embolism (PCE) group and non-pulmonary cement embolism (NPCE) group by comparing pre- and postoperative pulmonary CT scan images. The demographic and clinical information of the patients was obtained. Demographic data in the two groups were compared using the chi-square test for qualitative data and the unpaired t test for quantitative data. Multiple logistic regression analysis was used to identify risk factors related to pulmonary cement embolism.

**Results:**

Pulmonary cement embolism was detected in 11 patients (23.4%), and all patients were asymptomatic and followed up regularly. Risk analysis showed that multiple segments (≥3, p=0.022), thoracic vertebrae (p=0.0008), and unipedicular puncture approach (p=0.0059) were risk factors for pulmonary cement embolism. There was a high incidence of pulmonary cement embolism if bone cement leaked into the para vertebral venous plexus in the thoracic vertebra (p<0.0001). Vein leakage of cement was related to the integrity of the vertebral cortex.

**Conclusion:**

The number of involved vertebrae, lesion location, and puncture approach are independent risk factors for pulmonary cement embolism. There was a high incidence of pulmonary cement embolism if bone cement leaked into the para vertebral venous plexus in the thoracic vertebra. Surgeons should consider these factors when formulating therapeutic strategies.

## Introduction

The spine is the third common site of metastasis, following the lung and the liver (1). It has been reported that approximately 60–70% of patients with systemic cancer will have spinal metastasis (2). Spinal metastases can cause spinal instability, pathological fractures and spinal cord compression, all of which severely affect the quality of life of patients, shortening their lifespans (3). With advancements in cancer treatment, including radiotherapy, molecular targeted therapy, and immunotherapy, the survival rate of cancer patients has greatly improved, which amplifies the problems caused by spinal metastases (4).

Minimally invasive techniques have shown great advantages in the treatment of spinal metastases without causing severe neurological deficits (5). Among these techniques, percutaneous vertebroplasty (PVP) is most commonly used. It involves the percutaneous injection of an acrylic cement, polymethylmethacrylate (PMMA), into the vertebrae under image guidance (6). Vertebroplasty can relieve pain by stabilizing the compromised vertebrae, and the pain alleviation rate has been reported to be up to 70-94% (7–9). However, vertebroplasty has a very limited effect on the control of tumor progression (10).

Radiofrequency ablation (RFA) is another minimally invasive treatment for spinal metastases. It focuses a high-frequency alternating current through a needle electrode into surrounding tissues under image guidance, resulting in friction heating and tissue necrosis (11). RFA was first applied in the musculoskeletal system to treat osteoid osteoma in 1992 (12), and now it has been widely used to treat spinal metastases (13). The combined use of vertebroplasty and RFA has been proven to be safe and effective in treating spinal metastases for stabilisation and pain relief (14, 15).

Pulmonary cement embolism (PCE) is a rare complication of vertebroplasty. The incidence is reported to vary from 0.3% to 28.6% in patients with osteoporosis fracture who have undergone PVP or PKP (16–21). RFA is thought to decrease the risk of PCE (15, 22, 23). It cause thrombosis of the venous plexus, which may prevent embolization events during cement injection (22, 24, 25). An animal study found that a layer of dense cord can be formed at the edge of the tumor after RFA, and this biomembrane barrier can prevent bone cement leakage into the spinal canal during PVP (26). However, there is no related research on PCE in spinal metastatic patients following PVP and RFA.

In this study, we report our experience with vertebroplasty and RFA in the treatment of spinal metastases and analyze the risk factors for PCE following the operation, aiming to help develop therapeutic strategies and prevent this complication.

## Materials and methods

### Basic information

Spinal metastatic patients treated in the Department of Orthopedics of the Cancer Hospital of the Chinese Academy of Medical Science between February 2021 and February 2022 were retrospectively enrolled, and 47 patients were included in this analysis. All patients gave written informed consent, and the study was approved by the ethics board committee of the hospital.

The demographic and clinical information of patients were obtained from electronic medical records, including age, sex, diagnosis, and involved vertebrae. The visual analog scale (VAS) score (0 = no pain, 10 = severe pain) was recorded to evaluate pain intensity. The Spinal Instability Neoplastic Score (SINS) was calculated per published guidelines (27). Plain radiographs and CT scans of the corresponding vertebrae were obtained before and after the operation. If patients received systemic treatment (chemotherapy, targeted therapy, immunotherapy, endocrinotherapy, bone-protecting agents, and so on) or radiation before surgery was also recorded.

### Inclusion criteria and contraindications

The enrollment criteria included the following: i) diagnosis of metastatic cancer; ii) clinical and imaging evidence (MRI or CT) of vertebral metastases in the cervical, thoracic, lumbar or sacral segments; iii) pulmonary CT before and after the operation; iv) expected survival time >3 months; and v) PVP combined with RFA.

Contraindications for the procedure included: i) clinical signs of spinal cord compression or cauda equina syndrome; ii) fractures with epidural involvement and contact with spinal cord or nerve roots; iii) the lesions close to the vital structures such as nerves, spinal cord, blood vessels; and iv) local infection at the puncture site or septicemia. Relative contraindications included vertebral body height reduced more than 75%, and transient chemotherapy−induced hematologic anomalies, including leukopenia (<2.5×10^9^/L), thrombocytopenia (<100.0×10^9^/L) and elevated international normalized ratio >1.5.

### Operative procedures

The patients were placed in the prone position with conscious sedation. A puncture trocar (Zhongshan Shiyitang Medical Equipment Co., Ltd, China) was inserted from the vertebral pedicle to the anterior one-third of the vertebral body under the guidance of C-arm fluoroscopy (Siemens Healthcare, Munich, Germany). The trocar was removed, and a biopsy device (STERYLAB, Italy) was placed to obtain the bone fragments for pathology. Then, a monopole RFA electrode (17G) (MedSphere Shanghai, China) was inserted through the cannula. RFA was conducted for 10 min at a temperature ranging from 80°C to 100°C. The electrode was removed, and prepared high-viscosity polymethyl methacrylate (PMMA) bone cement (Weigao Medical GmbH, China) was injected into the vertebral body under intermittent fluoroscopic examination from the lateral plane. Injection was stopped when substantial resistance was met, or when the PMMA cement reached the posterior margin of the vertebral body, or cement extravasation was identified through fluoroscopy (**Supplementary Figure 1**). For multi-level cases, single cement kit was used for each level. 40mg methylprednisolone was given before radiofrequency ablation.

### Postoperative management

The patients were sent back to the ward after the operation and made to lie in bed for 6 hours, then could move freely, but a spinal brace was advised. Vital signs as well as sensory motor functions of the lower limbs were closely monitored. Plain radiographs and CT scans were performed before discharge.

The patients were followed up every 3 months for at least 9 months. Pulmonary CT scans were not necessary unless the patient complained of pulmonary-related symptoms. However, patients with spinal metastasis are always hospitalized several times for radiotherapy, chemotherapy, or other treatments, and pulmonary CT scans are essential for hospitalization. PCE was confirmed by comparing pre- and postoperative pulmonary images.

### Statistical analysis

Statistical analysis was performed with Prism 8 software (GraphPad Software, San Diego, CA, USA). All measurement data are described as the mean and standard deviation (SD). Demographic data in both groups were compared using the chi-square test for qualitative data and the unpaired t test for quantitative data. Multiple logistic regression analysis was carried out to identify risk factors that were significantly related to PCE resulting from cement leakage. A p value < 0.05 was considered statistically significant.

## Results

### Baseline characteristics of the patients

PVP and RFA were performed for 47 patients with a total of 84 segments, including 34 thoracic vertebrae and 50 lumbar vertebrae (**Table 1**). The patients were 19 males and 28 females with a mean age of 59.9 ± 1.6 years (range 34 to 81 years). The preoperative VAS score was 5.1 ± 0.3, and the preoperative SINS score was 9.6 ± 0.4. Among the 47 patients, 42 patients showed osteolytic lesions, and 5 patients exhibited osteoblastic lesions. Operations were performed on one segment in 27 patients, two segments in 11 patients, three segments in 7 patients, four segments in 1 patient, five segments in 2 patients, and six segments in 1 patient. 28 patients received systemic treatment before surgery, and 14 vertebral levels in 8 patients got pre-operative radiation treatment. Among the 84 vertebrae, pathologic compression fracture was found in 35 segments, and the other 49 segments had no compression fracture with obvious imaging abnormalities. The amount of cement injected per lesion ranged from 1.5 to 12 ml with a mean volume of 6.1 ± 0.2 ml. The postoperative pathological diagnoses of the spinal tumors confirmed that they were all metastatic tumors: 12 from lung cancer, 12 from breast cancer, 5 from kidney cancer, 4 from prostate cancer, 2 from bile duct cancer, 2 from thyroid cancer, 2 from gastric cancer, 1 from liver cancer, 1 from ovarian cancer, 1 from cervical cancer, 1 from esophageal cancer, 1 from soft tissue Ewing’s sarcoma and 3 from unknown malignancies (**Table 2**).

**Table 1:** Baseline characteristics of the patients.

	n (%)
Number of patients	47
Age (years)	59.9 ± 1.6
Sex	
Male	19 (40.4)
Female	28 (59.6)
VAS	5.1 ± 0.3
SINS	9.6 ± 0.4
Systermic treatment	
Yes	28 (59.6)
No	19 (40.4)
Preoperative radiation	
Yes	8 (17.0)
No	39 (83.0)
Spine lesion type	
Osteolytic	42 (89.4)
Osteoblastic	5 (10.6)
Segment number	
Single	27 (57.4)
Two	11 (23.4)
Three	5 (10.6)
Four	1 (2.1)
Five	2 (4.3)
Xix	1 (2.1)
Total number of vertebrae affected	84
Thoracic vertebra	34 (40.5)
Lumbar vertebra	50 (59.5)
Vertebrae with pathological fracture	
Yes	35 (41.7)
No	49 (58.3)
PMMA volume per level (ml)	6.1 ± 0.2

**Table 2:** Pathologic diagnosis of 47 patients.

Primary tumor	n (%)
lung cancer	12 (25.5)
breast cancer	12 (25.5)
kidney cancer	5 (10.6)
prostate cancer	4 (8.5)
bile duct cancer	2 (4.3)
thyroid cancer	2 (4.3)
gastric cancer	2 (4.3)
liver cancer	1 (2.1)
cervical cancer	1 (2.1)
ovarian cancer	1 (2.1)
esophageal cancer	1 (2.1)
Ewing’s sarcoma	1 (2.1)
unclear	3 (6.4)
Total	47

### Analysis of the risk factors for pulmonary cement embolism

According to the detection of postoperative pulmonary CT scans, the patients were divided into pulmonary cement embolism group (PCE group) and non-pulmonary cement embolism group (NPCE group). The presence of cement in the pulmonary arteries was identified in 11 patients (incidence rate 23.4%), including 5 males and 6 females, with a mean age of 55.7 ± 3.9 years. There were 36 patients in the NPCE group, including 14 males and 22 females, with a mean age of 60.5 ± 1.6 years (**Table 3**).

**Table 3:** Comparison of risk factors for PCE between patients of two groups.

	NPCE	PCE	p
Number of patients	36	11	
Age (years)	60.5 ± 1.6	55.7 ± 3.9	0.203
Sex			0.698
Male	14	5	
Female	22	6	
VAS	5.1 ± 0.3	5.1 ± 0.6	0.994
SINS	9.7 ± 0.5	9.1 ± 1.0	0.542
Systermic treatment			0.698
Yes	22	6	
No	14	5	
Spine lesion type			0.354
Osteolytic	33	9	
Osteoblastic	3	2	
Segment number			0.022
≥3	3	4	
≤2	33	7	

**Figure 1:**
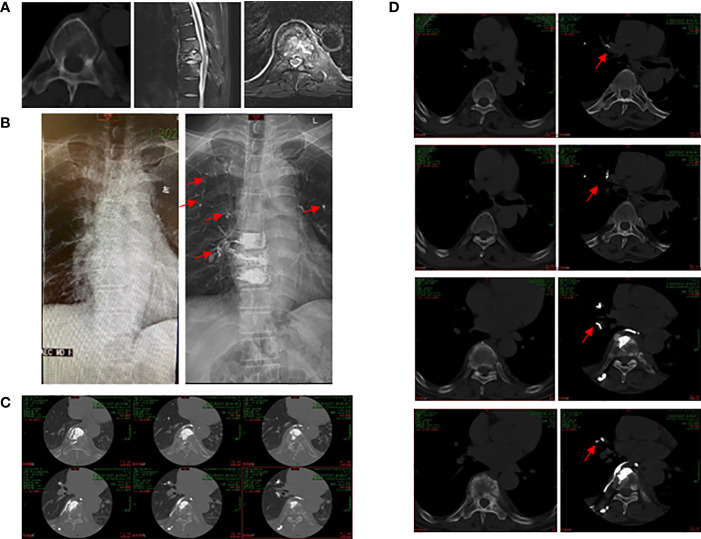
51 years old male patient with spinal metastasis from lung cancer underwent PKP and RFA. **(A)** Pre-operative radiographs showed an obvious lesion of eighth thoracic vertebral body. **(B)** Pre-operative (left) and post-operative (right) X-ray images of the chest. Cement pulmonary embolus (red arrows) were found in both lungs after operation. **(C)** Post-operative CT scan illustrated that the cement embolus leaked into the perivertebral venous system. **(D)** Pre-operative (left) and post-operative (right) CT scan images. Red arrows showed the cement embolus.

All the patients in the PCE group were asymptomatic. No dyspnea, chest pain, cough, tachycardia, or hypoxia was observed. The patients were followed up regularly. There were no critical patients who needed surgical treatment or died. In all 11 patients, pulmonary emboli were located in the subsegmental or peripheral arteries, and no central pulmonary artery embolism was found (**Figure 1**).

No significant differences were found in age, sex, preoperative VAS scores, SINS scores, or if got systemic treatment between the patients in the NPCE group and PCE group (**Table 3**). Among the 11 PCE patients, 9 patients showed osteolytic destruction, and the other 2 patients showed osteoblastic lesions, which was not significantly different from the NPCE group. However, 4 patients (36.4%) had multiple segments (≥3) involved in the PCE group, while only 8.3% of patients (3 patients) had multiple segment involvement in the NPCE group, which was a significant difference (p=0.022).

We then set the individual vertebrae as the study object. Lesion location, cortex integrity, compression fracture, preoperative radiation, vein leakage, and PMMA injection volume in vertebrae of the two groups were analyzed (**Table 4**). The 11 PCE patients had 25 segments involved in total, including 17 thoracic vertebrae and 8 lumbar vertebrae. The NPCE patients had 59 segments in total, including 17 thoracic vertebrae and 42 lumbar vertebrae. The results revealed that patients with thoracic vertebrae treated had a greater chance of developing pulmonary cement embolism (p=0.0008). Patients who had cement vein leakage during surgery also had a significantly increased risk compared with those who did not (p=0.017), especially when leakage occurred in the thoracic vertebrae (p<0.001). The two groups did not show significant differences in cortex integrity, compression fracture, of if got preoperative radiation.

**Table 4:** Risk factors for PCE in terms of individual vertebra.

	NPCE	PCE	p
Total number of vertebrae	59	25	
Lesion location			0.0008
Thoracic vertebra	17	17	
Lumbar vertebra	42	8	
Cortex integrity			0.586
Disrupted	34	16	
Intact	25	9	
Compression fracture			0.098
Yes	28	7	
No	31	18	
Preoperative radiation			0.24
Yes	8	6	
No	51	19	
Vein leakage			0.017
Yes	21	16	
No	38	9	
Vein leakage location			<0.0001
Thoracic vertebra	0	15	
Lumbar vertebra	21	1	

### Parameters related to technical characteristics

The incidence of complications was closely related to the operators’ skills. The parameters related to technical characteristics including operative approach, PMMA volume per level, and operating time were analyzed. Among the 25 segments of PCE group, 80% were treated through unipedicular approach, while the proportion is 47.5% in NPCE group (p=0.006) (**Table 5**). As the volume of thoracic vertebrae was smaller than lumbar vertebrae, we separated volume on injection reporting between thoracic and lumbar vertebrae. However there were no significant difference between NPCE and PCE groups. There was no difference as to operating time of two groups either.

**Table 5:** Parameters related to technical characteristics.

	NPCE	PCE	p
Operative approach			0.006
Unipedicular	28	20	
Bipedicular	31	5	
PMMA volume per level (ml)			
Thoracic vertebra	5.2 ± 0.7	5.2 ± 0.4	0.999
Lumbar vertebra	6.6 ± 0.3	6.9 ± 0.6	0.682
Operating time per level (min)	77.1 ± 3.0	71.3 ± 3.2	0.256

### Relationship of vertebral cortex disruption to vein leakage of cement

Previous data showed that there was a high incidence of PCE if bone cement leaked into the paravertebral venous plexus. We next analyzed the risk factors related to vein leakage of cement. Among the 47 patients with 84 vertebrae, 37 vertebrae had vein leakage of bone cement (leakage group, 44%), and the other 47 segments did not have leakage (nonleakage group, 56%). Vertebra integrity, compression fracture, lesion type, location, and PMMA volume were analyzed (**Table 6**). The results showed no significant differences in lesion type, location, or PMMA volume between the two groups. However, 75.7% of segments (28) in the leakage group showed a disrupted cortex, and only 46.8% of segments (22) in the nonleakage group showed a significant difference (p=0.008).

**Table 6:** Analysis of the risk factors for vein leakage of cement.

	Nonleakage	Leakage	p
Total number of vertebrae	47	37	
Cortex integrity			0.008
Disrupted	22	28	
Intact	25	9	
Spine lesion type			
Osteolytic	41	27	0.098
Osteoblastic	6	10	
PMMA volume per level (ml)	7.8 ± 0.3	6.4 ± 0.3	0.2
Lesion location			0.183
Thoracic vertebra	22	12	
Lumbar vertebra	25	25	

## Discussion

Pulmonary cement embolism is a rare complication of cement augmentation, first reported by Padovani et al. (28) in 1999. Several retrospective studies reported that the incidence of PCE on osteoporosis patients varied from 0.3% to 28.6% (16–21). Spinal tumors are another surgical indication for vertebroplasty, especially spinal metastasis. The risk of PCE in patients with spinal metastasis is thought to be increased (29). Asem analyzed 78 cancer patients with malignant vertebral fractures who underwent vertebroplasty, PCE was detected in 10 (12.8%) patients (30). To my knowledge, we first reported the incidence of PCE in patients with spinal metastasis who undergo PVP with RFA. In our study, we analyzed 84 segments in 47 spinal tumor patients who underwent treatment. The incidence of PCE was 23.4% (11/47), which is higher than Asem’s study.

Risk factor analysis can help to reduce the incidence of PCE. Previous studies showed that number of treated vertebral levels, fracture location and operation timing, amount of PMMA injected were thought to be associated with PCE (20, 31). Asem’s study showed that multiple myeloma is associated with the highest risk, and no difference in incidence was observed between patients with osteoporotic or malignant vertebral fractures (30).

Our analysis revealed that involvement of a larger number of vertebrae was a factor and that thoracic vertebrae were associated with PCE, which is consistent with previous study. This is readily comprehensible. Thoracic vertebrae are closer to the heart and lung. Operating on a larger number of involved vertebrae will take more time and more PMMA cement will need to be injected, which is challenging not only for the patients but also for the surgeons. Therefore, operating on more than three segments at a time is not recommended (20). We also found the unipedicular approach has a high rate of PCE. However, a previous meta-analysis showed the incidences of cement leakage were similar between the bilateral PVA and unilateral PVA groups (32), which is contrary to our results. In our experience, in order to get the same stabilization effect, we usually injected more volume of bone cement through unipedicular approach compared to the cement volume of each side through bipedicular approach. The injection pressure of each puncture channel may also be high, which could induce cement leakage. Definitely this result was related to the operators’ experience, and the explanation was just a hypothesis. We need to identify it in the future.

Our results also revealed that there was a high incidence of PCE if bone cement leaked into the paravertebral venous plexus, especially when occurred in the thoracic vertebrae. Risk factors for vein leakage of cement have been well studied, including involved segments and surgical skills (33–36). Our data indicated that vertebral cortex integrity is related to vein leakage of cement. If the bone cortex is disrupted, it is easier for the cement to pass through the cortex or into the paravertebral venous plexus.

All patients were followed up for at least 9 months, and no patients developed severe pulmonary embolism. Pulmonary CT scans showed that the emboli were stable and did not enlarge. For PCE, there are still no standard treatment guidelines (37). Asymptomatic patients may need close clinical monitoring (38). Some scholars have suggested that anticoagulation should be used to prevent progressive pulmonary artery occlusion; however, there is no consensus on the specific timing anticoagulant treatment (39, 40). For PCE patients with severe symptoms, surgical removal of the embolus and anticoagulant therapy are recommended (41–43).

There are several limitations to this study. The primary limitation is that this was a single-center, retrospective study, including a certain selection bias. Some patients did not undergo postoperative pulmonary CT, which may have led to the underestimation of asymptomatic patients. A secondary limitation is that all operations were performed by the surgical team, operators’ skills play a pivotal role in the development of PCE, we did not analyze the different operators or their operative habits.

## Conclusion

Our results showed that the number of involved vertebrae, lesion location, and puncture approach are independent risk factors for PCE. There was a high incidence of PCE if bone cement leaked into the para vertebral venous plexus in the thoracic vertebra. The relative risk factors should be fully considered when implementing therapeutic strategies to prevent the occurrence of PCE.

## Data availability statement

The raw data supporting the conclusions of this article will be made available by the authors, without undue reservation.

## Ethics statement

The studies involving human participants were reviewed and approved by National Cancer Center/National Clinical Research Center for Cancer/Cancer Hospital, Chinese Academy of Medical Sciences and Peking Union Medical College. The patients/participants provided their written informed consent to participate in this study. Written informed consent was obtained from the individual(s) for the publication of any potentially identifiable images or data included in this article.

## Author contributions

The authors’ contributions to this study were as follows: SY and LW contributed to the study design. LW, ML contributed to the data collection. LW, XZ, ZZ, XL, TL and LX contributed to the statistical analysis. LW and ML played the main role in writing the manuscript. All authors contributed to the article and approved the submitted version.
