# Supplementary material for: Risk factors for pulmonary cement embolism after percutaneous vertebroplasty and radiofrequency ablation for spinal metastases
Source: Front Oncol. 2023 May 5;13:1129658. doi: 10.3389/fonc.2023.1129658 (PMC10196379; doi:10.3389/fonc.2023.1129658)
Supplement: Supplementary file 1 [file Image_1.pdf]

Supplementary figure 1

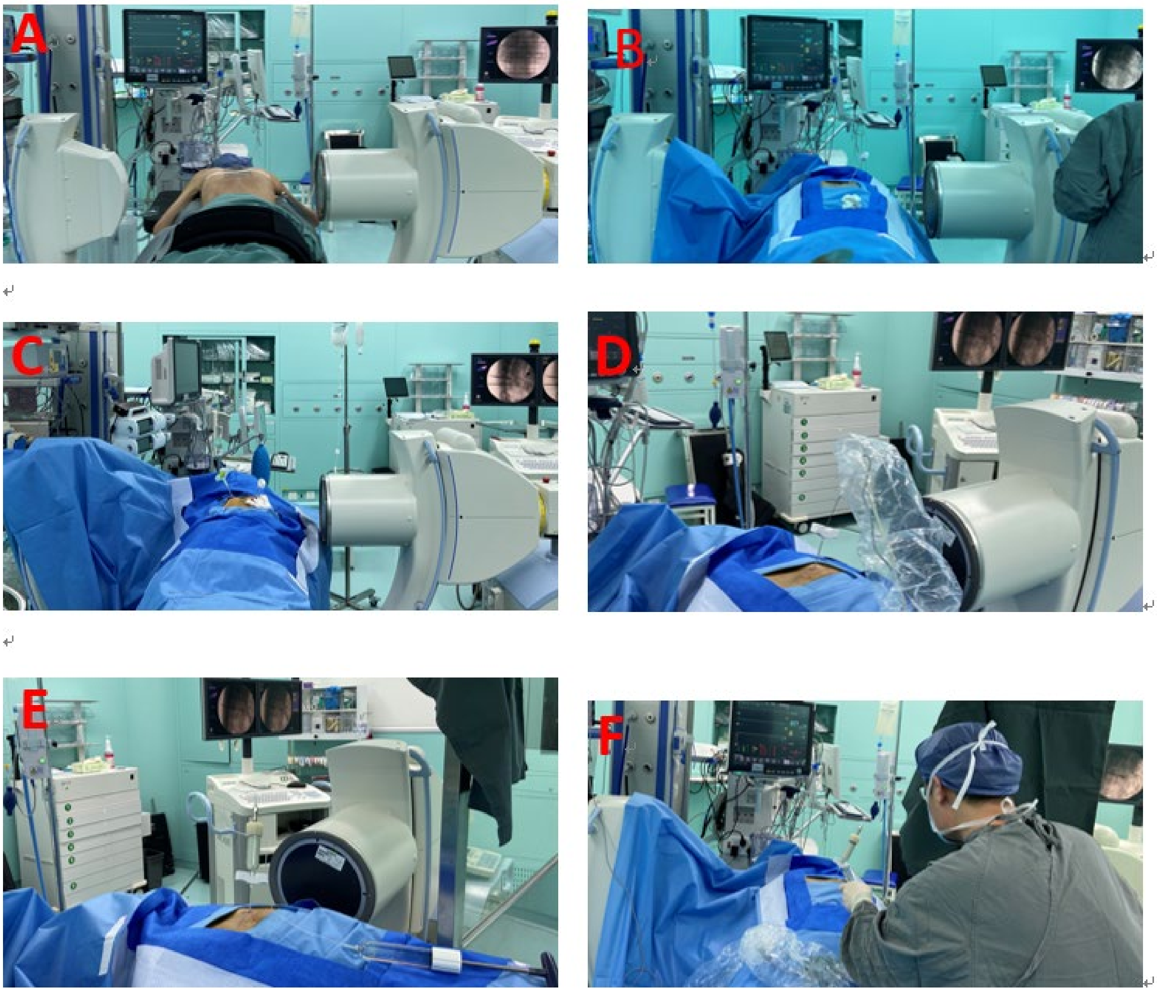

The images of surgery procedure (not from the same patient).

A: Patient was placed in the prone position. The operative level was identified under the guidance of C-arm fluoroscopy.

B: Puncture trocar was inserted from the vertebral pedicle to the anterior one-third of the vertebral body under the guidance of C-arm fluoroscopy.

C: A biopsy device was placed to obtain the bone fragments for pathology.

D: A monopole RFA electrode (17G) was inserted through the cannula.

E: Bone cement pusher filled with prepared high-viscosity polymethyl methacrylate bone cement was inserted into the vertebra.

F: Bone cement was injected into the vertebral body under intermittent fluoroscopic examination from the lateral plane.
